# Evaluation of functional deficits using the Ankle‐GO™ score before and after arthroscopic anatomic lateral ankle ligament reconstruction among patients suffering from chronic ankle instability

**DOI:** 10.1002/ksa.70296

**Published:** 2026-01-26

**Authors:** Kinan Freiha, Ronny Lopes, Mohamad K. Moussa, Eugénie Valentin, Gauthier Rauline, François Fourchet, Brice Picot, Alexandre Hardy

**Affiliations:** ^1^ Clinique du Sport Paris Paris France; ^2^ Department of Orthopedic Surgery Groupe Hospitalier Sélestat‐Obernai Sélestat France; ^3^ Centre Orthopédique Santy, FIFA Medical Center of Excellence, Hôpital Privé Jean Mermoz, Groupe Ramsay Lyon France; ^4^ Physiotherapy Department La Tour Hospital Swiss Olympic Medical Centre Meyrin Switzerland; ^5^ French Society of Sports Physical Therapist (SFMKS Lab) Pierrefitte‐sur‐Seine France; ^6^ Inter‐University Laboratory of Human Movement Sciences Chambéry France

**Keywords:** Ankle‐GO™, arthroscopic anatomic lateral ankle ligament reconstruction, chronic ankle instability, psychological readiness, return to sport

## Abstract

**Purpose:**

To evaluate the evolution of ankle deficits in patients suffering from chronic ankle instability (CAI) following an arthroscopic anatomic lateral ankle ligament reconstruction (AALALR) based on Ankle‐GO™.

**Methods:**

This prospective cohort (2022–2023) included patients suffering from CAI who underwent an AALALR. The primary outcome was the evolution of ankle deficits at 5 months postsurgery, assessed using the Ankle‐GO™, which includes the modified Star Excursion Balance Test (mSEBT), Figure‐of‐8 Test (F8T), Side Hop Test (SHT) and Single‐Leg Stance Test (SLS), the Foot and Ankle Ability Measure (Foot and Ankle Ability Measure—Activity of Daily Living [FAAM‐ADL] and Foot and Ankle Ability Measure—Sport [FAAM‐Sport]) and the Ankle Ligament Reconstruction–Return to Sport after Injury (ALR‐RSI) scale. Secondary outcome was the return to sports (RTS). Factors influencing lower postoperative Ankle‐GO^TM^ were evaluated.

**Results:**

Fifty patients (mean age 29.8 ± 11.1 years) were included. The Ankle‐GO™ score increased significantly from 8.2 ± 4.7 to 13.9 ± 5.4 at 5 months postoperatively (*p* < 0.01; *r* = 0.80). Among the individual components, ALR‐RSI showed the greatest improvement, rising from 28.0% to 69.0% (*p* < 0.01; *r* = 0.86), followed by FAAM‐Sport (56.5%–80.1%; *p* < 0.01; *r* = 0.71). The FAAM‐ADL score increased significantly from 82.4% to 91.8% (*p* < 0.01). The F8T did not improve significantly (*p* > 0.05). At 5 months, 35 patients (70.0%) had returned to sport, although only 11 (31.4%) had resumed at their pre‐injury level. Preoperative Ankle‐GO™ was lower in patients not returning to sport (6.4 vs. 9.1; *p* = 0.04).

**Conclusion:**

This short‐term study confirmed that the Ankle‐GO™ score effectively tracks recovery following AALALR. The score reflects improvements in both static and dynamic stability as well as psychological readiness. Additionally, preoperative scores were found to predict a patient's ability to RTS.

**Level of Evidence:**

Level II, prospective cohort study.

**Abbreviations**
AALALR: arthroscopic anatomic lateral ankle ligament reconstruction
ALR‐RSI: Ankle Ligament Reconstruction–Return to Sport after Injury scale
ANT: anterior
CAI: chronic ankle instability
F8T: Figure‐of‐8 Test
FAAM‐ADL: Foot and Ankle Ability Measure—Activity of Daily Living
FAAM‐Sport: Foot and Ankle Ability Measure—Sport
LAS: lateral ankle sprain
MRI: magnetic resonance imaging
mSEBT: modified Star Excursion Balance Test (mSEBT)
PL: posterolateral
PM: posteromedial
RTS: return to sport
SHT: Side Hop Test
SLS: Single‐Leg Stance Test

## INTRODUCTION

Ankle sprains are the most frequent sports injuries [47]. Despite the recommendation of the International Ankle Consortium for evaluation and treatment of lateral ankle sprain (LAS) [8, 13], and the advanced rehabilitation protocols and programmes [30], around 40% of patients will develop chronic ankle instability (CAI). It is referred to as recurrent sprains, persistent pain and giving way perception [14, 21]. Progression of CAI can lead to a permanently painful ankle and early development of arthritis [20].

Conservative treatment with a tailored rehabilitation programme targeting each specific deficit around the ankle remains the gold standard for CAI [8, 10, 11]. In case of failure, surgical intervention becomes necessary, with numerous techniques being described [6, 11]. Arthroscopic anatomic lateral ankle ligament reconstruction (AALALR) was widely adopted and showed successful outcomes [16, 17, 27, 28]. In addition to lateral ligament reconstruction, arthroscopic technique allows for ankle joint exploration, reduced rate of infection and immediate treatment of any associated intra‐articular pathologies [15, 25, 44].

While AALALR has demonstrated successful outcomes, evaluation often relies on subjective patient‐reported outcome measures (PROMs) such as the Karlsson–Peterson score or the Cumberland Ankle Instability Tool (CAIT) [7, 26, 42, 46, 48, 49]. However, those studies lack important elements for the evaluation of the ankle deficits, such as objective evaluation of the functional status and a psychological assessment of the readiness to return to sports (RTS) [35, 41]. Picot et al. developed and validated the Ankle‐GO™ for a global assessment of the ankle, which has excellent psychometric properties with good discriminant and predictive value for RTS following LAS [36]. Recently, Hardy et al. proved its validity for the ankle evaluation following AALALR [19].

The purpose of our study was to track the evolution of ankle deficits following AALALR for patients suffering from CAI using an evidence‐based tool, the Ankle‐GO™. The secondary aims were to describe the rate and level of RTS at 5 months postoperatively, to determine whether preoperative variables were associated with insufficient functional improvement and to assess whether preoperative Ankle‐GO™ scores could predict RTS at the same or higher level.

Our hypothesis was that Ankle‐GO™ would increase significantly after AALALR.

## MATERIALS AND METHODS

### Study design and inclusions

A prospective multicenter cohort study was performed among patients who underwent AALALR at two centres from January 2022 to January 2023.

AALALR was indicated for patients suffering from CAI, defined in accordance with the International Ankle Consortium recommendations [14]. This included a history of at least one significant ankle sprain and a perceived sensation of giving way refractory to at least 6 months of conservative treatment [14, 15]. Each patient underwent a thorough preselection examination focused on assessing ankle laxity, including the anterior drawer test and lateral tilt assessment. An imaging protocol was mandatory to confirm the diagnosis, which included standard ankle X‐rays (anteroposterior, lateral and mortise views) to rule out bony abnormalities, stress radiographs confirming mechanical instability (pathological laxity compared to the healthy contralateral side), a Meary's line radiograph to assess hindfoot alignment [24] and ankle magnetic resonance imaging (MRI) to confirm the rupture of the anterior talofibular ligament and calcaneofibular ligament as well as concomitant chondral injuries [32].

The exclusion criteria encompassed patients who underwent other types of reconstruction, as well as those who received a ligament repair. Patients requiring revision surgery or additional procedures for related injuries, such as osteochondral lesions of the talus or fibular tendinopathy, were also excluded. Additionally, patients with osteoarthritic changes classified as Takakura Stage 2 or higher, or a sedentary lifestyle who did not engage in sports, were not included in the study.

This study was approved by the local institutional ethics committee. Following the decision for surgical intervention, each patient provided their informed consent to participate in the study.

### Surgical technique and rehabilitation protocol

All patients underwent surgery under spinal anaesthesia, performed by senior surgeons according to the technique described by Guillo et al. and later refined by Lopes et al. [18, 27]. The procedure began with harvesting the gracilis tendon; if the tendon was already harvested, an allograft was prepared as a substitute [16, 27].

The procedure begins with harvesting the gracilis tendon or preparing the allograft, both of which are then tagged at both ends and soaked in a vancomycin solution [40]. An arthroscopic exploration of the tibio‐talar joint through the usual anteromedial and anterolateral portals was exhibited. Calcaneal, talar and fibular tunnels were drilled in their anatomical position. The transplant was first secured to the talus using a bio‐tenodesis screw. One end of the tendon graft was first secured to the talus using a bio‐tenodesis screw, and the other end was passed through the calcaneal tunnel. After that, the tendon graft was then pulled into the fibula with the adjustable button and finally fixed in valgus in the calcaneus using bio‐tenodesis screw as well [18].

After surgery, patients were placed in a walking boot for 3 weeks, to be worn both day and night. Full weight‐bearing was permitted right away. Passive rehabilitation, including range of motion and drainage exercises, began early, with active rehabilitation starting after 3 weeks.

### Ankle‐GO^TM^ composite score

**Figure 1:**
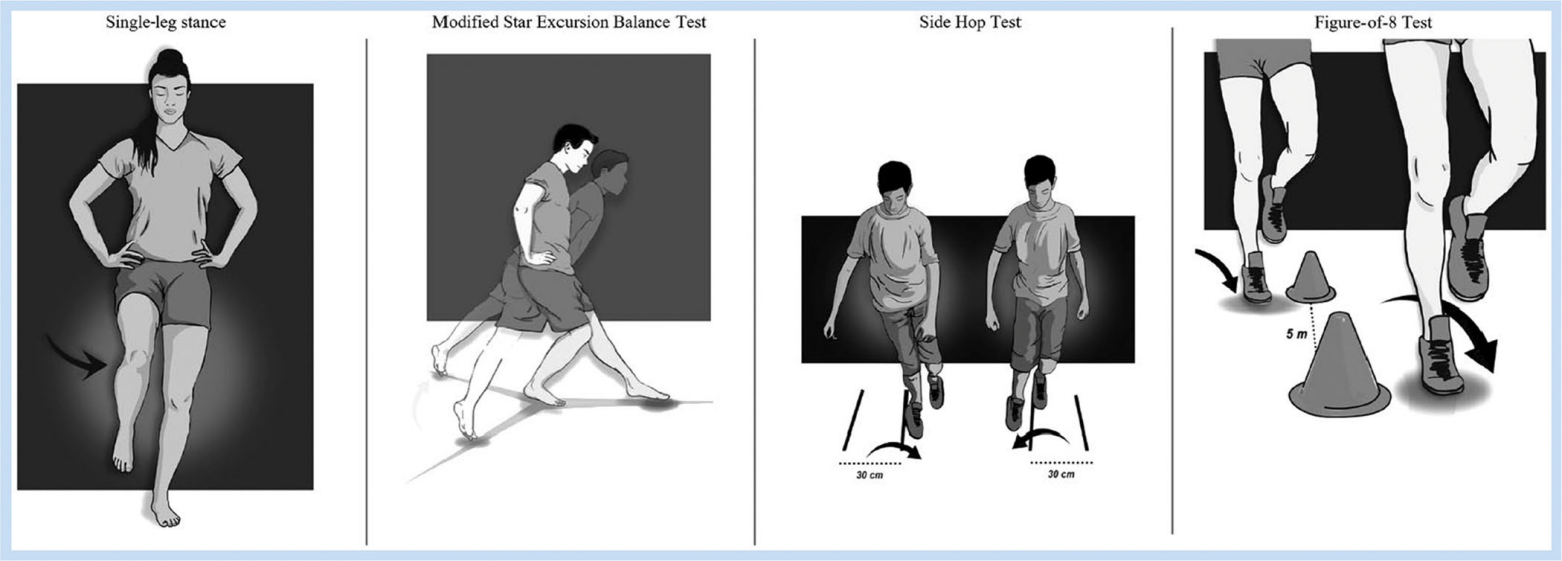
Representation of the functional performance tests. *Source*: Adapted from Picot et al. [36].

Ankle‐GO^TM^ is a composite score based on the sum of four functional tests and two patient self‐reported questionnaires, each weighted according to its level of evidence. The overall score ranges from 0 to 25, with a higher score indicating greater functional capability [36]. The four functional tests [35], as shown in Figure 1, include:

1. Modified Star Excursion Balance Test (mSEBT): The patient stands barefoot on the tested foot at the centre of a ‘Y’ shape formed by three lines. They are required to reach as far as possible with the opposite leg in three directions: anterior (ANT), posteromedial (PM) and posterolateral (PL), before returning to the starting position [31]. The ANT direction primarily reflects dorsiflexion mobility, whereas PL and PM directions reflect neuromuscular control and abductor/adductor strength.
2. Side Hop Test (SHT): evaluates hopping performance and frontal‐plane dynamic stability by having the patient perform 10 rapid lateral hops across two lines 30 cm apart [9].
3. Figure‐of‐8 Test (F8T): assesses multiplanar plyometric ability and agility. The patient skips in a figure‐8 pattern around two posts that are 5 m apart, aiming to complete the task as quickly as possible [5].
4. Single‐Leg Stance Test (SLS): measures static postural control. The patient stands on the injured side with one foot on a firm surface, hands on hips, knee slightly flexed and eyes closed. They must maintain this position for 20 s. Any loss of balance is counted as an error [39].

After each test, patients were asked if they felt any instability; a response of ‘NO’ resulted in an additional point being awarded. In addition to these tests, two self‐reported questionnaires contribute to the overall score. The first is the Foot and Ankle Ability Measure (FAAM), which includes two subscales: the Daily Living Activities subscale (FAAM ADM), composed of 21 items and the Sports subscale (FAAM sport), consisting of 8 items [3, 29, 45]. The second is Ankle Ligament Reconstruction—Return to Sport after Injury (ALR‐RSI): Assesses the psychological readiness for returning to sports [1, 43].

Appendix 1 presents the detailed scoring system of the Ankle‐GO™, outlining the weighting and calculation method for each of its components to clarify how the total score out of 25 points is derived.

### Outcome measures

The primary outcome measure was the functional status of the ankle, assessed using the Ankle‐GO™ score, recorded 1 month preoperatively and 5 months postoperatively. The 5‐month postoperative assessment was selected as it was considered to correspond to the transitional phase, where patients are typically cleared to begin progressive RTS activities. This timing was deemed appropriate to evaluate functional deficits at the specific moment when RTS decisions were being made [22].

Several specific analyses were conducted for this outcome, including the comparison of pre‐ and postoperative Ankle‐GO™ scores for each patient and across the full sample. This also included a detailed analysis of each component of the score to explore specific improvements in dynamic and static postural control, multiplanar plyometric ability, functional capacity and psychological readiness. The study also explored potential risk factors associated with poor functional improvement, defined as a gain of less than the minimal detectable change (MDC) reported in the literature [36]. The MDC for the Ankle‐GO™ was set at 5.6 points [36]. Potential risk factors analysed included age, sex, side of injury, type of sport (categorized as pivot with contact, pivot without contact or in‐line) and sport level (classified as recreational, occasional, competitor or professional).

The secondary outcome measures were the RTS rate and the level of return at 5 months following surgery. Patients were categorized based on whether they had returned to their pre‐injury sport, and if so, whether it was at the same or lower level.

### Data collection

Data were collected prospectively through an internet‐based software, Websurvey, which was accessed and filled out by surgeons for physical examination and surgical technique and by patients for demographic characteristics and questionnaires.

An experienced physical therapist, independent of the surgical team and blinded to the surgical details, evaluated the ankle and entered each patient's Ankle‐GO™ score into the dedicated app ‘https://anklego.com/login’. Five months postoperatively, the same therapist reperformed the assessment. At this stage, patients respond to a ‘YES’ or ‘NO’ question to confirm whether they have returned to sports. If they have resumed sports, they specify their performance level by selecting one of the following options: ‘same’ or ‘inferior’. This assessment reflected the patient's subjective perception of their performance by comparing it preoperatively.

### Statistical analysis

Means and standard deviations were reported for descriptive statistics of quantitative variables, while frequencies and proportions were provided for qualitative variables.

To compare preoperative and 5‐month postoperative scores, the Wilcoxon signed‐rank test was used, as it is appropriate for non‐normally distributed paired data.

In addition, effect sizes (*r*) were calculated to assess the magnitude of the differences observed.

The effect size was computed using the formula *r *= $\frac{Z}{\surd N}$, where *Z* is the standardized test statistic from the Wilcoxon test, and *N* is the number of paired observations. Effect sizes were interpreted using common thresholds: small effect: *r* < 0.3; moderate effect: *r* < 0.5 and large effect: *r* ≥ 0.5.

A *p* value ≤ 0.05 was considered statistically significant. All statistical analyses were conducted using R software (version 4.2).

## RESULTS

### Demographic characteristics

A total of 50 patients with a mean age of 29.8 (±11.1) years were included. The study population consisted of 21 males (42.0%) and 29 females (58.0%). Twenty‐five patients (50.0%) practiced a high level of sports (professional and competitive), while the remaining 25 played recreational or occasional sports (50.0%). Patient demographics and the distribution of the patient's usual sports are summarized in Table 1.

**Table 1:** Baseline characteristics of the study population.

	*N* = 50
Age	29.8 (11.1)
Sex	
Female	29 (58.0%)
Male	21 (42.0%)
Side	
Right	31 (60.8%)
Left	20 (39.2%)
Sport	
Pivot with contact	18 (36.0%)
Pivot	18 (36.0%)
In line	14 (28.0%)
Sport level	
Professional	23 (46.0%)
Competitor	2 (4.0%)
Occasional	2 (4.0%)
Recreational	23 (46.0%)

### Functional recovery as measured by the Ankle‐GO^TM^ score

**Figure 2:**
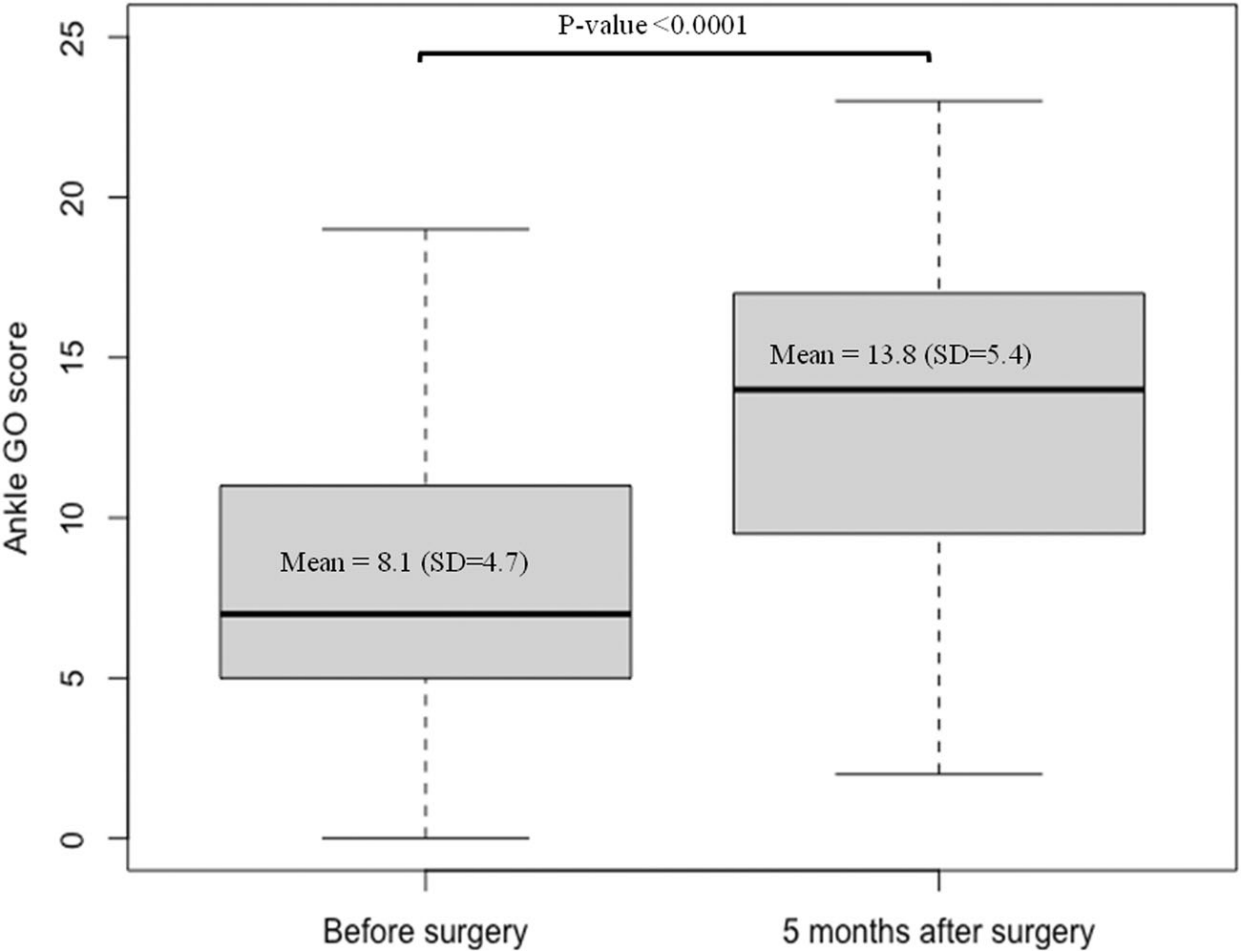
Box plot illustrating the progression of Ankle‐GO™ scores from the preoperative assessment to 5 months postoperatively. SD, standard deviation.

**Figure 3:**
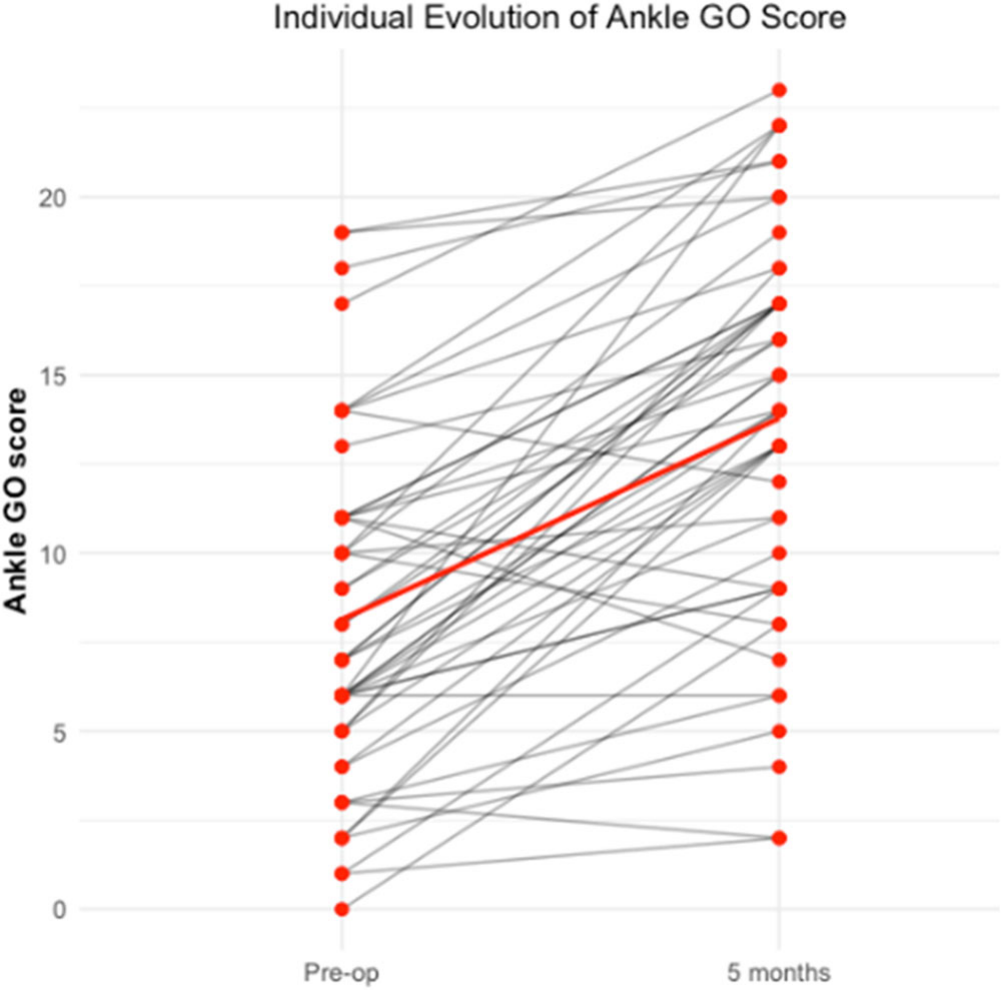
Individual evolution of Ankle‐GO™ scores between the preoperative assessment and 5 months postoperatively. Each line represents one patient's score progression.

The Ankle‐GO™ score significantly increased from a mean of 8.2 (standard deviation [SD] = 4.7) preoperatively to 13.9 (SD = 5.4) at 5 months postoperatively (*p* < 0.01), with a large effect size (*r* = 0.80) (Figure 2). The individual evolution of Ankle‐GO™ scores demonstrated a clear upward trajectory in most patients except five of them (Figure 3).

Except for the F8T, all components of the Ankle‐GO™ improved significantly, with the greatest changes observed in the ALR‐RSI (*r* = 0.86), FAAM‐Sport (*r* = 0.71), FAAM‐ADL (*r* = 0.69), SLS (*r* = 0.59), SHT (*r* = 0.45) and posterolateral mSEBT (*r* = 0.48) (Table 2).

**Table 2:** Ankle‐GO™ results preoperatively and 5 months postoperatively.

	Pre‐op, *N* = 50	5 Months, *N* = 50	Effect size	*p* value
mSEBT				
% norm COMP	82.5 (7.0)	84.2 (5.6)	0.26	0.04
% normANT	63.8 (7.5)	62.3 (6.1)	0.25	0.11
% normPM	93.4 (9.6)	95.0 (7.0)	0.16	0.16
% normPL	90.4 (10.1)	95.3 (7.7)	0.48	<0.01
SHT	17.4 (10.1)	14.3 (7.3)	0.45	<0.01
F8T	16.2 (7.1)	15.3 (5.5)	0.09	0.59
SLS	2.9 (2.6)	1.3 (1.7)	0.59	<0.01
FAAM				
% FAAM ADL	82.4 (13.2)	91.8 (11.9)	0.69	<0.01
% FAAM Sport	56.5 (21.1)	80.1 (18.2)	0.71	<0.01
ALR RSI	28.0 (18.4)	69.0 (21.3)	0.86	<0.01
Ankle‐GO™ score	8.2 (4.7)	13.9 (5.4)	0.80	<0.01

Abbreviations: Ankle‐GO™ score, global composite score of ankle function; F8T, Figure‐of‐8 Test; mSEBT, modified Star Excursion Balance Test; Pre‐op, preoperative; SHT, Side Hop Test; SLS, Single‐Leg Stance; % FAAM ADL, Foot and Ankle Ability Measure—Activities of Daily Living; % FAAM Sport, Foot and Ankle Ability Measure—Sports subscale; % normANT, normalized anterior reach; % norm COMP, normalized composite score; % normPL, normalized posterolateral reach; % normPM, normalized posteromedial reach; % RSI, Ankle Ligament Reconstruction—Return to Sport after Injury.

Dynamic stability, assessed through the mSEBT and the SHT, showed partial improvement. The composite mSEBT score increased slightly from 82.5% to 84.2% (*p* = 0.04) with a small effect size at *r* = 0.26. Among the three directions, only the posterolateral reach improved significantly (90.4%–95.3%; *p* < 0.01; *r* = 0.48), while the anterior and posteromedial directions remained stable (*p* = 0.11 and *p* = 0.16, respectively). In parallel, the SHT completion time improved from 17.4 to 14.3 s (*p* < 0.01; *r* = 0.45), indicating better frontal‐plane reactive stability and hopping control.

Static stability, measured by the SLS, improved significantly, with a reduction in error count from 2.9 to 1.3 (*p* < 0.01; *r* = 0.59). Multiplanar plyometric ability, assessed by the F8T, did not show meaningful improvement at 5 months. Completion time decreased marginally from 16.2 to 15.3 s, without reaching statistical significance (*p* = 0.59; *r* = 0.09).

Self‐reported function progressed markedly. The FAAM‐ADL increased from 82.4% to 91.8% (*p* < 0.01; *r* = 0.69), and the FAAM‐Sport from 56.5% to 80.1% (*p* < 0.01; *r* = 0.71). The ALR‐RSI showed the largest improvement, rising from 28.0% to 69.0% (*p* < 0.01; *r* = 0.86).

### Risk factors for failing to achieve 5.6‐point increase in Ankle‐GO^TM^ Score

No significant association was seen between age, sex, side of injury, type of sport and the level of performance with failing to achieve an improvement of the score (Table 3).

**Table 3:** Univariate logistic regression: Risk factors for failing to achieve 5.6‐point increase in Ankle‐GO™ Score.

	OR	95% CI	*p* value
Sex			
Female	1 ref	—	
Male	0.73	0.24, 2.27	0.59
Age	1.0	0.95, 1.05	0.83
Side of the injury			
Right	1 ref		
Left	0.32	0.09, 1.00	0.054
Categories of sport			
Pivot with contact	1 ref	—	
Pivot without contact	1.73	0.46, 6.89	0.42
In line	0.60	0.14, 2.44	0.48
Level of sport			
Occasional or regular	1 ref	—	
Professional or competition	1.29	0.42, 3.97	0.66

Abbreviations: CI, confidence interval; OR, odds ratio.

### RTS

At 5 months postoperatively, 35 out of 50 patients (70.0%) had returned to sport. Among them, only 11 patients (31.4%) were able to resume activity at their pre‐injury level, while the remaining 24 (68.6%) returned at an inferior level. Sixteen patients (31.4%) had not yet resumed sporting activity at the time of follow‐up. Preoperative Ankle‐GO™ scores were significantly lower in patients who had not returned to sport compared with those who had (6.4 vs. 9.1; *p* = 0.04) but were not significantly different between those returning at their pre‐injury level versus a lower level (7.9 vs. 9.2; *p* = 0.48) (Table 4).

**Table 4:** Association between return to sports and Ankle‐GO™ score before and after surgery.

	Return to sport		
	No	Yes (all levels)	*p* value
Ankle‐GO™ score before surgery, mean (SD)	6.4 (3.8)	9.1 (4.9)	0.04

	**No**	**Yes at pre‐injury level**	***p* value**
Ankle‐GO™ score before surgery, mean (SD)	7.9 (4.6)	9.2 (5.1)	0.48

Abbreviation: SD, standard deviation.

## DISCUSSION

### Main finding

This study demonstrates the suitability of the Ankle‐GO™ composite score for assessing functional recovery after AALALR. These results are consistent with the literature [19, 26, 36]. The mean score increased from 8.2 to 13.9, exceeding the MDC of 1.2 points established by Picot et al. in the original validation study [36]. While the Ankle‐GO™ was initially developed to guide RTS decisions after LAS, our results highlight its ability to capture meaningful recovery after surgical treatment for CAI. Notably, the average score at 5 months (13.9) surpasses the threshold of 11 points identified in a recent prospective study as being associated with a 12‐fold higher probability of becoming a ‘coper’ 1 year after LAS [33]. In that study, a coper was defined as a patient who had returned to their pre‐injury sport level without functional limitations or episodes of giving way over 12 months [33]. Although our cohort underwent surgical intervention rather than conservative treatment, achieving this benchmark early in the postoperative course suggests that AALALR can effectively restore functional stability. While scores such as the Karlsson–Peterson score and the CAIT are more commonly used and have established Minimal Clinically Important Differences (MCIDs), they primarily focus on subjective instability and pain [48, 49]. In contrast, the Ankle‐GO™ captures objective functional deficits. Our findings complement these traditional scores by demonstrating that functional recovery, as measured by Ankle‐GO™, aligns with the subjective improvements typically reported in the literature using CAIT and Karlsson–Peterson scores.

### Item analysis

Almost all components of the Ankle‐GO™ showed a significant improvement following surgery and rehabilitation, except for the figure‐of‐eight test. The most marked gain was observed in psychological readiness to RTS, with the ALR‐RSI increasing by 41.5% (*r* = 0.86), well beyond the MDC of 8.37% reported by Picot et al. [34]. These results align with Hardy et al., who reported ALR‐RSI scores of 60.7% and 75.9% at 4 and 6 months postoperatively, respectively [19].

Regarding functional recovery, the FAAM improved by 10 points for ADL and 23.6 points for the Sport subscale at 5 months postoperatively. These gains exceed the MCID reported by Martin et al. (8 points for ADL, 9 for Sport), reflecting meaningful recovery [29]. Picot et al. showed that patients who became copers after a LAS already had higher FAAM scores by 2 months [33]. In our study, results at 5 months postoperatively were slightly superior to those of the coper group in both the ADL (92.9 vs. 92) and Sports subscales (79.8 vs. 71.9), but inferior to those reached by Hertel and Corbett [21, 33]. Our results are consistent with Hardy et al., who reported similar FAAM scores postoperatively (90 and 69 at 4 months, 84 and 95 at 6 months) [19]. Importantly, our FAAM‐ADL scores clearly exceeded the threshold proposed by Gribble et al. for defining CAI (ADL < 90%), while the FAAM‐Sport score progressed to 79.8, approaching the threshold of Gribble et al. at 80% [14].

In terms of postural control, static balance showed notable improvement. The SLS error count decreased (*r* = 0.60), below the pathological threshold (≥3 errors) defined by Linens et al., consistent with findings of Hardy et al. 6 months postoperatively [19, 23]. When analysed in light of the performance of copers after LAS, these results can be interpreted as reaching the level of postural control typically expected in functionally recovered patients [33].

For dynamic stability, the SHT timing improved significantly (*r* = 0.46) but did not reach the MDC of 5 s described by Caffrey et al. [5]. However, when compared with the performance of copers reported by Picot et al. [33] and with the postoperative values described by Hardy et al. [19], this improvement appears consistent with meaningful functional recovery.

Regarding mSEBT, our study did not show an improvement in the posterolateral component, not surpassing the required MDC 97.6% defined by Picot et al. [37]. Those measurements correlate with those of Hardy et al., showing a similar composite score around 84%. In addition, our SEBT normCOMP % is better than that of the copers patients following LAS [33]. Overall, this reflects a relative improvement in dynamic postural control; however, a relative risk of lower limb injury remains present as demonstrated by Plisky et al. (SEBT normCOMP %<94%) [38]. Looking more closely, the PM composite—associated with an increased risk of LAS—exceeded the threshold identified by Attenborough et al. (>77.5%), corresponding to an odds ratio of 4.04 (95% confidence interval [CI]: [1.00, 16.35]) [2]. Moreover, the anterior component of the mSEBT remained stable in our study, consistent with the findings of Hardy et al., suggesting that dorsiflexion range of motion (DFROM) is not significantly affected postoperatively, and no stiffness was observed [12, 19].

Finally, the Figure‐of‐8 test showed no significant change, mirroring the plateau seen in both copers and postoperative patients in previous studies [5, 19, 33]. This test, which involves multiplanar plyometric effort, may require a longer timeline or more sport‐specific retraining to improve.

### RTS

RTS at 5 months was achieved in 70% of patients, aligning with the 75% rate at 6 months reported by Bouveau et al. and comparable to the findings of Hardy et al., who observed 45% at 4 months and 76.5% at 6 months [4, 19]. A notable difference was observed when comparing our results with those of Rupp et al., who reported a 96% RTS at 8.3 ± 6.2 months, and Li, who reported 95% in his systematic review [22, 41]. However, when examining in depth, multiple criteria may justify this discrepancy. The longer follow‐up (63.7 ± 28.0 months) and time to RTS (8.3 months in their study vs. 5 months in ours) might explain the difference [41]. Additionally, the study by Rupp et al. was conducted on a smaller sample size (23 participants vs. 50 in our study) and involved a more active population, where higher motivation could have played a role in achieving a faster RTS [41]. Moreover, a slight modification in the surgical technique was observed, which may have contributed to improved stability. Only 31% resumed sport at the pre‐injury level, which was lower than the 50% reported by Bouveau et al. [4]. This difference may be attributed to the longer follow‐up period in their study.

Those rates confirm the objectivity of the Ankle‐GO™ as a tool for assessing the ankle status and its correlation to RTS. Hence, a higher Ankle‐GO™ reflects a lesser ankle deficit, which will, indubitably, lead to a higher probability of RTS.

### Limitations

The small sample size and the lack of a control group are considered as limitations of this study. Better patient selection and an analysis of subgroups (recreational and professional) could provide more specific results. A longer follow‐up would be essential for a better assessment of the ankle by recording the Ankle‐GO™ and comparing it to a control group. Finally, while 50% of our cohort participated in competitive or professional sports, future studies focusing exclusively on professional athletes are warranted to better evaluate the specific recovery trajectories and return‐to‐sport timelines in this high‐demand population.

## CONCLUSION

This short‐term study showed that improvements after AALALR were consistently captured by the Ankle‐GO™ score. The score was able to demonstrate the restoration of static and dynamic stability, while also quantifying the boost in psychological readiness for RTS. Low Ankle‐GO™ score preoperatively predicted the ability to RTS.

## AUTHOR CONTRIBUTIONS

Kinan Freiha, Ronny Lopes, Alexandre Hardy, François Fourchet and Brice Picot designed the study. The preparation of the material, the writing of the report and the critical revision of the work were carried out by Kinan Freiha, Mohamad K. Moussa, Alexandre Hardy, Eugénie Valentin, François Fourchet, Ronny Lopes and Brice Picot. Data collection was carried out primarily by Kinan Freiha and Gauthier Rauline, with support from Mohamad K. Moussa. Statistical analysis was carried out by Eugénie Valentin. Kinan Freiha drafted the first version of the manuscript together with Brice Picot, and all authors critically reviewed later versions until all authors approved the final manuscript. Alexandre Hardy is the guarantor of the data in this study.

## CONFLICT OF INTEREST STATEMENT

Alexandre Hardy is a consultant for Arthrex, EUROS and FH. Ronny Lopes is a consultant for Arthrex and a consultant and developer for Serf Extremity and Implant Service Orthopédie. The remaining authors declare no conflict of interest.

## ETHICS STATEMENT

This study involves human participants and was approved by the ethics committee. Participants gave informed consent to participate in the study/collection of data before taking part.

## Data Availability

Data are available upon reasonable request.

## Appendix 1

List of tests and questionnaires used for the construction of the Ankle‐GO™ score and system to determine the points for each component.

**Table:**

Tests		Test value	Weight	Maximum score by test
Single leg stance test [SLS]:		>3 errors	0	3
		1–3 errors	1	
		0 error	2	
		No Fear	+1	
Star excursion balance test [mSEBT]		< 90%	0	7
		90–95%	2	
		>95%	4	
		Anterior >60 %	+1	
		Postero‐medial >90 %	+1	
		No Fear	+1	
Side hop test [SHT]		>13 s	0	5
		10–13 s	2	
		< 10 s	4	
		No Fear	+1	
Figure‐of‐8 test [F8T]		>18 s	0	3
		13–18 s	1	
		< 13 s	2	
		No Fear	+1	
Foot and Ankle Ability Measure	Activities of Daily Living [FAAMad]	< 90 %	0	2
		90–95 %	1	
		>95 %	2	
	Sport [FAAMsport]	< 80 %	0	2
		80–95 %	1	
		>95 %	2	
Ankle Ligament Reconstruction‐Return to Sport after Injury (ALR‐RSI)		< 55 %	0	3
		55‐63 %	1	
		63–76 %	2	
		>76 %	3	

Abbreviations: ALR‐RSI, ankle ligament reconstruction‐return to sport after injury; ANT, anterior; FAAM, foot and ankle ability measure; F8T, figure‐of‐8 test; mSEBT, star excursion balance test; PM, posteromedial; PROM, patient‐reported outcome measure; SHT, side hop test; SLS, single‐leg stance test.
